# Single fixed low-dose rituximab as induction therapy suppresses de novo donor-specific anti-HLA antibody production in ABO compatible living kidney transplant recipients

**DOI:** 10.1371/journal.pone.0224203

**Published:** 2019-10-23

**Authors:** Yusuke Tomita, Kazuhiro Iwadoh, Yuichi Ogawa, Katsuyuki Miki, Yojiro Kato, Kotaro Kai, Akihito Sannomiya, Ichiro Koyama, Kumiko Kitajima, Ichiro Nakajima, Shohei Fuchinoue

**Affiliations:** Department of Surgery, Kidney Center, Tokyo Women’s Medical University, Tokyo, Japan; Children’s Hospital Boston, UNITED STATES

## Abstract

This study was conducted to evaluate de novo donor-specific anti-human leukocyte antigen (HLA) antibody (dnDSA) production leading to antibody-mediated rejection (ABMR) after rituximab induction in non-sensitized ABO-compatible living kidney transplantation (ABO-CLKTx). During 2008–2015, 318 ABO-CLKTx were performed at the Department of Surgery III at Tokyo Women’s Medical University Hospital. To reduce confounding factors, we adopted a propensity score analysis, which was applied with adjustment for age, gender, duration of pretransplant dialysis, HLA mismatch count, preformed DSA, non-insulin-dependent diabetes mellitus, immunosuppressive treatment, and estimated glomerular filtration rate (eGFR) on postoperative day 7. Using a propensity score matching model (1:1, 115 pairs), we analyzed the long-term outcomes of 230 ABO-CLKTx recipients retrospectively. Recipients were classified into a rituximab-treated (RTX-KTx, *N* = 115) group and a control group not treated with rituximab (C-KTx, *N* = 115). During five years, adverse events, survival rates for grafts and patients, and incidence of biopsy-proven acute rejection (BPAR) and dnDSA production for the two groups were monitored and compared. All recipients in the RTX-KTx group received rituximab induction on preoperative day 4 at a single fixed low dose of 100 mg; the CD19^+^ B cells were eliminated completely before surgery. Of those recipients, 13 (11.3%) developed BPAR; 1 (0.8%) experienced graft loss. By contrast, of C-KTx group recipients, 25 (21.7%) developed BPAR; 3 (2.6%) experienced graft loss. The RTX-KTx group exhibited a significantly lower incidence of BPAR (*P* = .041) and dnDSA production (13.9% in the RTX-KTx group *vs*. 26.9% in the C-RTx group, *P* = .005). Furthermore, lower incidence of CMV infection was detected in the RTX-KTx group than in the C-KTx group (13.9% in the RTX-KTx group *vs*. 27.0% in the C-KTx group, *P* = .014). No significant difference was found between groups for several other factors: renal function (*P* = .384), graft and patient survival (*P* = .458 and *P* = .119, respectively), and the respective incidences of BK virus infection (*P* = .722) and leukopenia (*P* = .207). During five-year follow-up, single fixed low-dose rituximab therapy is sufficient for ensuring safety, reducing rejection, and suppressing dnDSA production for immunological low-risk non-sensitized ABO-CLKTx.

## Introduction

In 2002, we conducted ABO-incompatible living kidney transplantation (ABO-ILKTx) for the first time ever reported using an anti-CD20 monoclonal antibody, rituximab [1, 2]. Later, this strategy was extended to preoperative desensitization therapy comprising rituximab and plasma-exchange or double filtration plasmapheresis (DFPP) [3, 4]. Follow-up studies revealed that inclusion of a fixed low dose of rituximab in the preoperative regimen for ABO-ILKTx recipients yielded better long-term outcomes [5].

Reducing acute/active antibody-mediated rejection (AABMR) has a crucially important role in renal function in the early stage after kidney transplantation (KTx). Progressive lesions leading to chronic active antibody-mediated rejection (CABMR) have been recognized as a cause of graft failure and loss [6, 7]. Loupy and Lefaucheur recently demonstrated that merging approaches, including histologic phenotypes, donor-specific anti-human leukocyte antigen (HLA) antibody (DSA) production, and gene-based biomarkers, are necessary for improved diagnoses and therapies of AABMR [8].

The chimeric mouse–human monoclonal antibody, rituximab, was developed originally for poorly differentiated refractory or follicular CD20 positive B cell non-Hodgkin lymphoma [9]. Because of its fewer associated side-effects and long-lasting effects, rituximab has been used against B cell immunity in organ transplantation, including induction therapy of the preoperative desensitization protocol for patients at high risk for immunological complications and treatment of AABMR after KTx [10]. Although antibodies produced by plasma cells are regarded as an important cause of ABMR, rituximab has no effect on hematopoietic stem cells, progenitor B cells, plasma cells, or existing antibodies in peripheral blood. However, rituximab has been regarded as targeting memory B cells and suppressing T-cell-mediated antigen presentation through B cells [11]. Some earlier reports of studies investigating ABO-ILKTx have described that rituximab might play an important role in preventing the reemergence of preexisting DSA and in reducing de novo DSA (dnDSA) after KTx [12].

Recently, anti-HLA antibodies, especially dnDSA after KTx, have been reported as strongly associated with AABMR and CABMR, leading to poor graft survival [13–17]. Solid-phase assays such as Luminex cross-match are currently capable of detecting low DSA levels more effectively than cell-based or membrane-based assays such as complement-dependent cytotoxicity cross-match and flow cytometry cross-match [18]. To prevent ABMR of the transplanted kidney, dnDSA after KTx must be reduced or eliminated. During short-term follow up, rituximab induction plus maintained standard immunosuppression were shown to be useful strategies for ABO-compatible KTx (ABO-CKTx) recipients [19–21]. Nevertheless, 45–70% kidneys included among these data were obtained from deceased donors. In addition, the association between long-term renal function and suppression of dnDSA by rituximab induction in immunologically low-risk living KTx remains questionable. This retrospective study was conducted to evaluate five-year outcomes of non-sensitized ABO-compatible living KTx (ABO-CLKTx) treated with a fixed low-dose rituximab as a part of induction therapy.

## Materials and methods

### Population

During January 2008 through December 2015, 318 ABO-CLKTx were performed at the Department of Surgery III at Tokyo Women’s Medical University Hospital. Patients were classified into two groups: one using rituximab induction (RTX-KTx group, *N* = 131) and a control group without rituximab induction (C-KTx group, *N* = 187). For this study, we used a single fixed low dose of rituximab (100 mg) as an induction protocol with standard immunosuppression for ABO-CLKTx. We excluded KTx from deceased donors and pediatric KTx from this study. The mean follow-up period was 4.085±1.194 years. All study participants provided informed consent. The appropriate ethics committee of the Tokyo Women’s Medical University approved the study design (#180509). Our study was conducted according to the principles of the Declaration of Helsinki.

### Propensity score analysis

We adopted a propensity score analysis (PSS) to ascertain the effects of RXM induction therapy on the development of biopsy-proven acute rejection (BPAR) in the 318 ABO-CLKTx. The occurrence of BPAR was selected as the objective variable in the PSS with the following 10 confounding factors as explanatory variables: donor and recipient ages, donor and recipient genders, duration of pretransplant dialysis, HLA-mismatch count in class I and II, preformed DSA, non-insulin-dependent diabetes mellitus, steroid early withdrawal, and estimated glomerular filtration rate (eGFR) on postoperative day 7. Using PSS method, the observed and the control groups were selected respectively from 131 RTX-KTx group members and 181 C-KTx group members. As a consequence, 115 pairs of RXM-KTx and C-KTx were chosen with a similar propensity score in each pair to develop BPAR, irrespective of rituximab induction therapy. Then we analyzed the outcomes of 230 ABO-CLKTx recipients retrospectively. During five years, survival rates for grafts and patients, incidence of BPAR and dnDSA production, and adverse events for the two groups were monitored and compared.

### Immunosuppressive regimen

The basic immunosuppressive regimen was basiliximab (BXM) induction, with cyclosporine or tacrolimus, mycophenolate mofetil (MMF), and methylprednisolone. On the day of surgery and on postoperative day 4, BXM was administered intravenously at 20 mg/day. Cyclosporine or tacrolimus, and MMF were started respectively on day 7 before surgery at 2 mg/kg/day or 6 mg/kg/day and 2000 mg/day to maintain blood trough levels. Methylprednisolone was administered intravenously at 250 mg on the day of surgery. Subsequently, it was tapered until discontinuation. All patients in the RTX-KTx group received rituximab on preoperative day 4 at a single fixed dose of 100 mg.

### Definitions and assignments of rejection

Suspected rejections were defined as serum creatinine increases of 25–30% compared to baseline with a rapid urine volume decline. We used allograft biopsy to diagnose AABMR, CABMR, and T-cell mediated rejection (TCMR). Kidney allograft biopsies were processed for light microscopy and immunofluorescence including C4d staining in all cases. Pathological findings were graded based on the Banff 2015 classification [22]. The histopathological scores were recorded as follows: interstitial inflammation (i) and tubulitis (t) (i + t); intimal arteritis (v), microvascular inflammation (glomerulitis (g) + peritubular capillaritis (ptc)); interstitial fibrosis (ci) and tubular atrophy (ct) (ci + ct); transplant glomerulopathy (cg); arteriosclerosis (cv); and C4d deposition on peritubular capillaritis. In addition, AABMR was defined as follows: 1) microvascular inflammation (g>0) or arteritis (v>0) in glomeruli; and 2) C4d positivity in peritubular capillaritis (ptc>0). We defined CABMR as follows: 1) chronic tissue injury (cg>0) or arterial intimal fibrosis; and 2) C4d positivity in peritubular capillaritis (ptc>0) or moderate microvascular inflammation (g+ptc≥2). We defined TCMR as follows: IA) significant interstitial inflammation (i2 or i3) and moderate tubulitis (t2); IB) significant interstitial inflammation (i2 or i3) and severe tubulitis (t3); IIA) mild intimal arteritis (v1) and/or interstitial inflammation; and IIB) severe intimal arteritis (v2) and/or interstitial inflammation. All patients with AABMR and CABMR were treated with methylprednisolone pulses (500 mg × 2 days) and rituximab (100 mg) with DFPP (2–3 times) or intravenous immunoglobulin (20 g × 3 days). Cases of TCMR were treated with methylprednisolone pulses and antithymocyte globulin (ATG) (1.5 mg/kg/day × 4–5 days).

### Diagnosis of adverse events

We monitored CMV viremia using CMV pp65 C10/C11 antigenemia in serum. We defined CMV infection and disease as positivity (> 10) for CMV pp65 C10/C11 antigenemia, with treatment using ganciclovir or valganciclovir. All CMV seronegative recipients who had received a kidney transplanted from a CMV seropositive donor received antiviral prophylaxis with valganciclovir for 6 months after KTx. We defined BK virus (BKV) infection as positive (> 1.0 × 10^2^ copy/m^3^) for BKV DNA in the plasma and urine, and conducted diagnosis by allograft biopsy. Leukopenia was defined as less than 2000/mm^3^ (Grades 3 and 4). It was treated with granulocyte-colony stimulating factor (G-CSF).

### Typing of HLA-antigens

#### Screening assays for anti-HLA antibodies

Before and after transplantation, all patients were screened using flow panel reactive antibodies (PRA) (One Lambda; Thermo Fisher Scientific Inc.) to identify the presence or absence of class 1 and/or class 2 anti-HLA IgG antibodies. The secondary antibody was added: FITC conjugated anti-human IgG antibody (One Lambda; Thermo Fisher Scientific Inc.). The sera were analyzed using FACS caliber flow cytometry (Becton Dickinson). The PRA were regarded as positive when a bead shifted more than 10% to the right of the negative control bead, and when multimodal staining was observed. All patients with anti-HLA antibodies underwent testing for HLA-DSA in sera.

#### SAFB assays

All patients with anti-HLA antibodies were tested using LABScreen SAFB (One Lambda; Thermo Fisher Scientific Inc.) to ascertain the HLA-DSA concentrations. After 20 μl of serum was added to 5 μl of class 1 panel or class 2 panel antigen beads, phycoerythrin-conjugated goat anti-human IgG (One Lambda; Thermo Fisher Scientific Inc.) was used as the second antibody. The mixture was processed according to the manufacturer’s instructions. We used SAFB covering HLA-A/B/DR/DQ antigens to assess the presence of anti-HLA antibodies. Patient sera were evaluated (LABScreen 100; Luminex Corp., Austin, TX). The manufacturer provided positive and negative control sera and beads for the respective tests. LABScreen SAFB were inferred as positive when the mean fluorescence intensity (MFI) value was higher than 500. Follow-up HLA-DSA levels were tested every year if serum creatinine was elevated 20–25% compared to baseline and/or proteinuria was detected after KTx.

### Statistical analyses

We performed PPS using software (JMP^®^ Pro 13; SAS^®^ Institute Inc., SAS Campus Drive, Cary, North Carolina USA). Other statistical analyses were conducted using another software package (Mathematica^®^ ver. 11; Wolfram Research Inc., 100 Trade Center Drive, Champaign, Illinois, USA). Results were expressed as mean ± SD. For statistical analyses, discrete data were tested using chi-square testing. Continuous variables were compared using Mann–Whitney U tests to compare two independent variables. Fisher’s exact test was used when frequencies of some cells were lower than 5. Freedom from BPAR and survival rates for grafts and patients were calculated using Kaplan–Meier estimation and were compared using Log-Rank tests. The average levels of eGFR for five years were compared using two-way ANOVA analysis of variance. Differences in survival rates between the two groups were assessed using log-rank tests. Differences for which a *P*-value of less than .05 was obtained were inferred as statistically significant.

## Results

### Patient characteristics

Baseline descriptive characteristics of 115 pairs are presented in Table 1. The final study population of recipients predominantly included male. The mean ages in the RTX-KTx group and the C-KTx group were, respectively, 58.5±8.9 and 59.1±11.0 (*P* = .228). The most common underlying kidney disease was non-insulin-dependent diabetes mellitus; approximately 20% of patients had undergone preemptive KTx. The mean durations of pretransplant dialysis in the RTX-KTx group and the C-KTx group were, respectively, 3.9±6.8 and 3.8±5.4 (*P* = .198). In the RTX-KTx group, all patients were treated with rituximab (*P*< .001). The donors were predominantly female. Mean ages in the RTX-KTx group and the C-KTx group were, respectively, 47.9±13.5 and 48.3±12.3 (*P* = .834).

**Table 1:** Patient characteristics.

	RTX-KTx	C-KTx	
*N*	115	115	*P*
Recipient			
Age (yr)	58.5±8.9	59.1±11.0	.228
Gender (male/female)	73/42	79/36	.403
Cause of ESRD			
Diabetes (IDDM)	4	8	.375**
(NIDDM)	28	29	.878
CGN	12	17	.320
IgAN	14	18	.445
NS	7	7	1**
PKD	5	4	1**
FSGS	3	1	.622**
Other	44	31	.067
Preemptive	24	20	.502
Times of KTx (1st/2nd)	111/4	111/4	1**
Duration of pretransplant dialysis (yr)	3.9±6.8	3.8±5.4	.198
HLA-mismatch			
AB (0/1/2/3/4)	14/30/43/20/8	10/35/42/20/8	.900
DR (0/1/2)	18/73/24	18/78/19	.688
Preformed DSA	2	1	1**
Immunosuppression			
Rituximab	115	0	< .001**
BXM	115	115	1**
CyA/TAC + MMF+ MP	44	44	1
CyA/TAC + MMF*	71	71	1
CMV serologic status			
Donor positive, Recipient negative	12	17	.320
Donor			
Age (yr)	47.9±13.5	48.3±12.3	.834
Gender (male/female)	40/75	36/79	.574

mean±SD

* steroid withdrawal;

** Fisher’s exact test

BXM, basiliximab; CyA, cyclosporine; TAC, tacrolimus; MMF, mycophenolate mofetil; ESRD, end-stage renal disease; IDDM, insulin-dependent diabetes mellitus; NIDDM, non-insulin-dependent diabetes mellitus; CGN, chronic glomerulonephritis; IgAN, IgA nephropathy; NS, atheronephrosclerois; PKD, polycystic kidney disease; FSGS, focal segmental glomerulosclerosis; KTx, Kidney transplantation; DSA, donor-specific antibodies

### Trends of B cells and maintained immunosuppression

**Fig 1:**
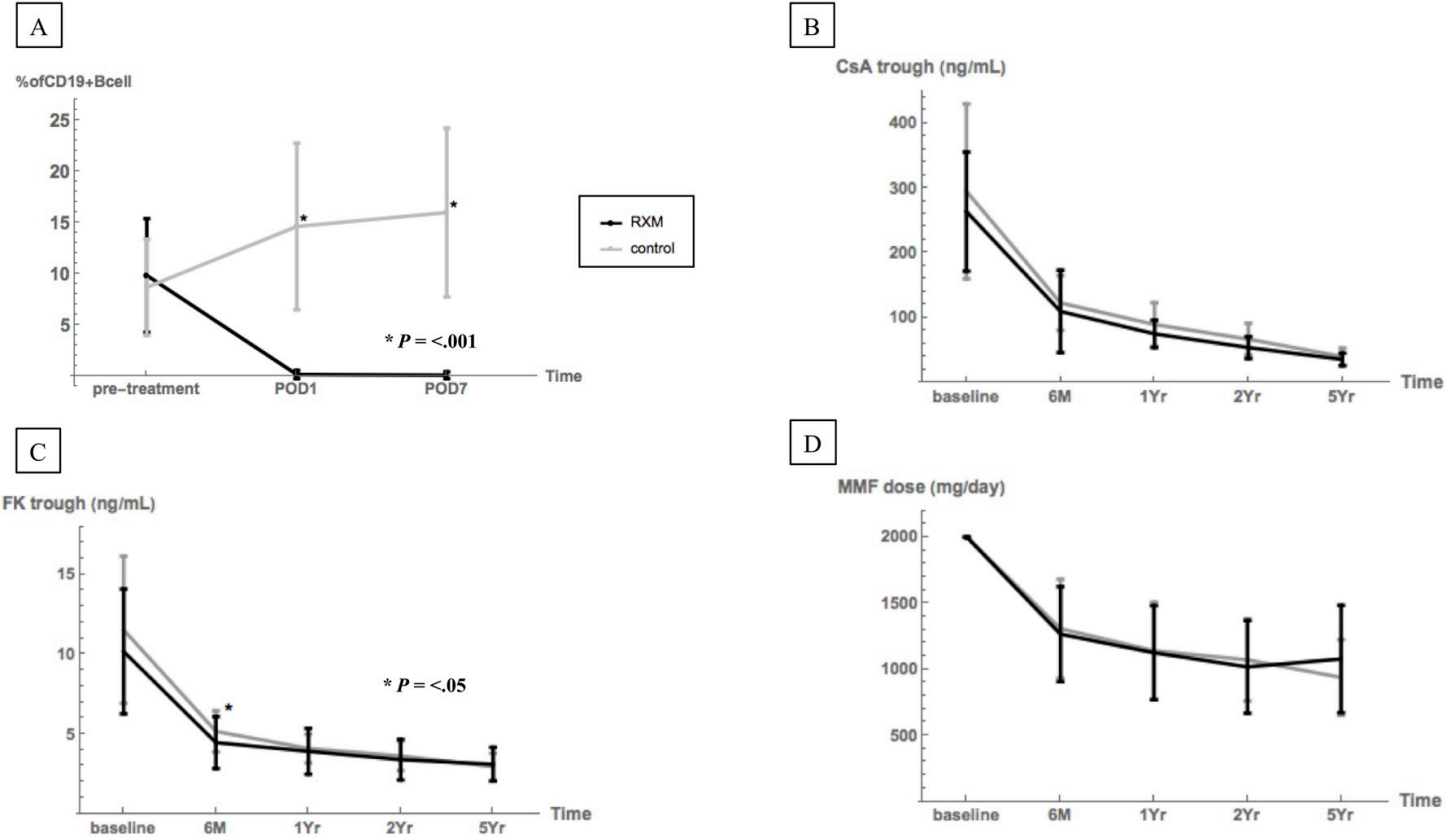
Trends of B cells and maintained immunosuppression. **(A)** The percentage of CD19^+^ B lymphocytes in peripheral blood treated with a single fixed low-dose rituximab injection (100 mg). **(B–D)** Trough levels of CyA (B) and TAC (C), and the dose of MMF (D) at baseline, 1 year, 2 years, and 5 years after KTx. The black line shows data of the rituximab-treated group (RTX-KTx group). The gray line shows data of control group, which was not treated with rituximab (C-KTx group).

To assess the effects of a 100 mg dose of rituximab as induction, we analyzed the percentage of CD19 positive B cells in peripheral blood mononuclear cells during pre-treatment and post-KTx (Fig 1A). Although the CD19 positive B cells in the RTX-KTx group were significantly depleted after rituximab treatment, those in the C-KTx group were slightly more numerous after KTx (*P*< .001). During the five-year follow up, no significant difference between the two groups was found in the trough levels of cyclosporine or tacrolimus. The dose of MMF maintained immunosuppression, except for the trough level of tacrolimus at 6 months after KTx (Fig 1B–1D).

### Death-censored graft survival and patient survival

**Fig 2:**
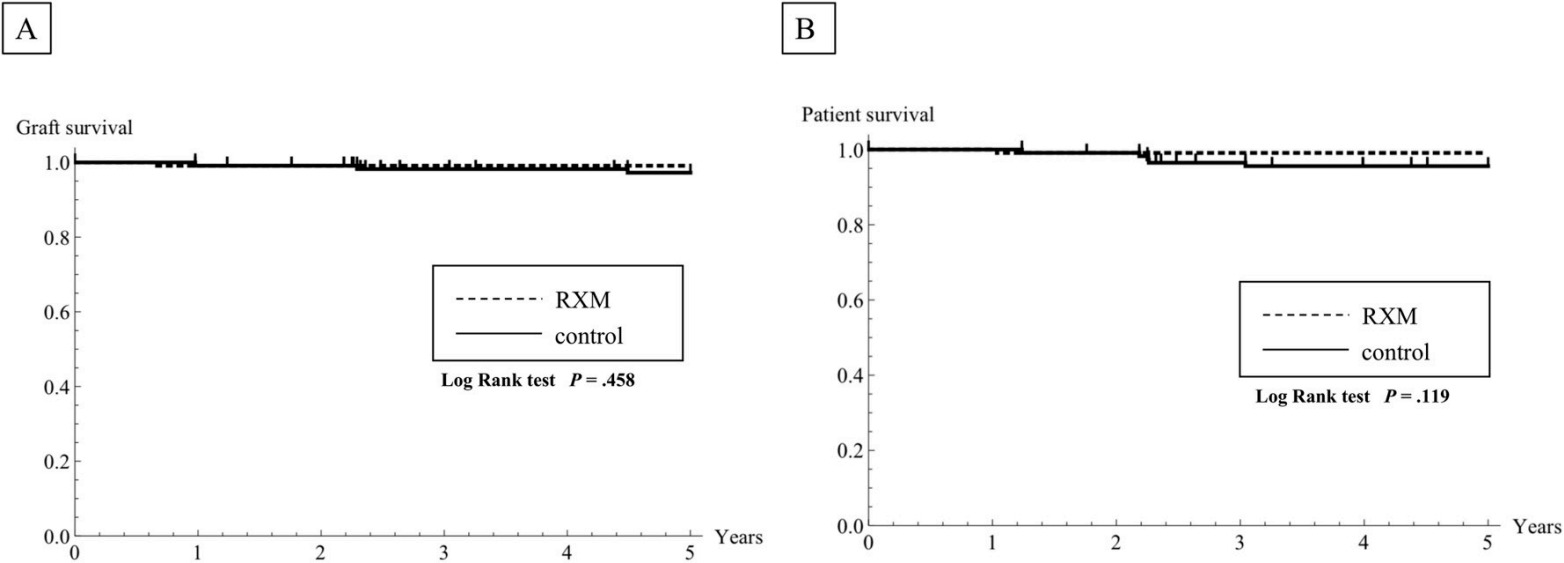
Kaplan–Meier estimation of five-year death-censored graft and patient survival between the rituximab-treated group (RTX-KTx group) and the control group (C-KTx group). No significant difference was found between groups. Comparisons were made using log-rank tests: *P* = .458, death-censored graft survival; and *P* = .119, patient survival. The dotted line shows data of the RTX-KTx group. The solid line shows data of the C-KTx group.

Of the 115 cases in the RTX-KTx group, 1 (0.7%) sustained graft loss because of ureteral stenosis of graft at 6 months after KTx. Death-censored graft survival rates in the RTX-KTx group and the C-KTx group were, respectively, 99.1% and 99.1% at one year, 99.1% and 98.2% at two years, and 99.1% and 97.2% at five years after transplantation (Fig 2A). Additionally, 1 patient (0.7%) in the RTX-KTx group died of cerebrovascular disease with a functioning kidney graft at 13 months after KTx. Patient survival rates in the RTX-KTx group and the C-KTx group were, respectively, 100% and 100% at one year, 99.1% and 99.1% at two years, and 99.1% and 95.5% at five years after transplantation (Fig 2B). No significant difference was found between the two groups with regard to the incidence of delayed graft function (DGF), eGFR and death-censored graft and patient survival rates for five years after transplantation (*P* = 1, *P* = .384, *P* = .458, and *P* = .119, respectively) (Table 2, Fig 2).

**Table 2:** Outcomes of post-KTx.

	RTX-KTx	C-KTx	
*N*	115	115	*P*
DGF (%)	1 (0.8)	2 (1.7)	1*
eGFR (mL/min/1.73m^2^)			.384
6 month	43.2±8.0	43.7±6.5	
1 year	45.6±9.5	53.3±13.6	
2 year	42.8±7.0	41.4±8.9	
5 year	35.1±21.5	39.0±10.8	
Graft loss (%)	1 (0.8)	3 (2.6)	.622*
Graft survival rates (%)			.458
1 year	99.1	99.1	
2 year	99.1	98.2	
5 year	99.1	97.2	
Death (%)	1 (0.8)	5 (4.3)	.213*
Patient survival rates (%)			.119
1 year	100	100	
2 year	99.1	99.1	
5 year	99.1	95.5	
Adverse events (%)			
CMV infection	16 (13.9)	31 (27.0)	.014
BKV infection	5 (4.3)	3 (2.6)	.722*
Leukopenia	30 (26.1)	22 (19.1)	.207

mean±SD

* Fisher’s exact test

DGF, delayed graft function; eGFR, estimated glomerular filtration rate

### Incidence of adverse events

Viral infections of CMV and BKV were observed in 16 cases (13.9%) in the RTX-KTx group, in 31 cases (27.0%) in the C-KTx group (*P* = .014), in 5 cases (4.3%) in the RTX-KTx group, and in 3 cases (2.6%) in the C-KTx group (*P* = .722). Although the incidence of severe leukopenia was observed in higher frequencies in the RTX-KTx group (26.1%), no significant difference was found between the two groups (*P* = .207) (Table 2).

### Incidence of BPAR

**Fig 3:**
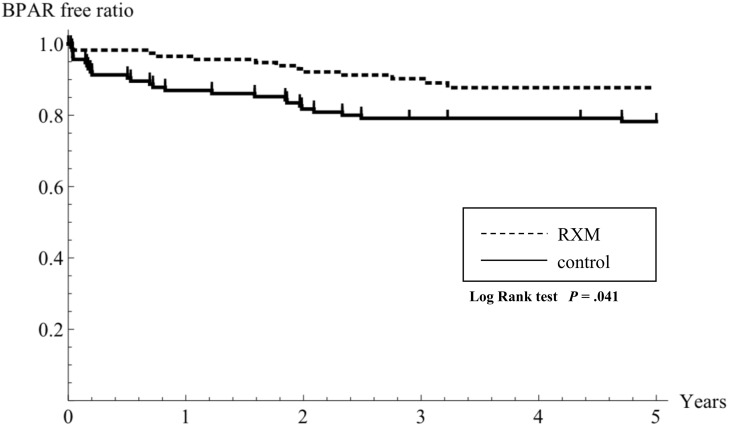
Kaplan–Meier estimation of the BPAR-free ratio in the rituximab-treated group (RTX-KTx group) and the control group (C-KTx group). The BPAR incidence was significantly lower in the RTX-KTx group. Comparisons were made using log-rank tests: *P* = .041. The dotted line shows data of the RTX-KTx group. The solid line shows data of the control group, which was not treated with rituximab.

The patients of overall BPAR in the RTX-KTx group and the C-KTx group were, respectively, 13 (11.3%) and 25 (21.7%) (*P* = .033) (Table 3). The proportions of patients without BPAR in the RTX-KTx group and the C-KTx group were, respectively, 96.5% and 86.9% at one year, 92.1% and 81.7% at two years, and 88.7% and 78.2% at five years after transplantation. The proportion of patients without such episodes was significantly lower in the RTX-KTx group (*P* = .041) (Table 3, Fig 3). Of all BPAR, a significantly lower proportion of TCMR was found for the RTX-KTx group (*P* = .018). However, no significant difference was found between the two groups with regard to the incidence of AABMR, CABMR, or mixed type rejection (*P* = .499, *P* = 1, and *P* = 1, respectively). Data of histopathological scores at BPAR diagnosis were similar in the two groups (Table 3).

**Table 3:** Incidence of rejection.

	RTX-KTx	C-KTx	
*N*	115	115	*P*
BPAR free ratio (%)			.041
1 year	96.5	86.9	
2 year	92.1	81.7	
5 year	88.7	78.2	
Total (%)	13 (11.3)	25 (21.7)	.033
AABMR	2	6	.499*
CABMR	6	5	1*
TCMR	3	11	.018*
IA	0	4	
IB	1	1	
IIA	2	3	
IIB	0	3	
Mixed type rejection	2	3	1*
AABMR+TCMR	1	1	
CABMR+TCMR	1	2	
Histopathological scores			
i+t	2.3±1.9	2.3±1.9	.658*
v	0.2±0.4	0.5±0.7	.436*
g+ptc	2.9±1.26	2.1±1.5	.225*
ci+ct	1.9±1.5	0.9±1.2	.088*
cg	0.6±1.0	0.2±0.4	.225*
cv	0	0.1±0.4	1*
c4d	1.2±0.9	0.8±1.0	.610*

* Fisher’s exact test

BPAR, biopsy-proven acute rejection; AABMR, acute antibody-mediated rejection; CABMR, chronic antibody-mediated rejection; TCMR, T cell-mediated rejection

### dnDSA production

The patients of overall dnDSA in the RTX-KTx group and the C-KTx group were, respectively, 16 (13.9%) and 31 (26.9%) (Table 4). The proportion of patients with dnDSA was significantly lower in the RTX-KTx group (*P* = .005). The majority of dnDSA observed in this study was HLA class II in both groups: 15 (93.7%) of 16 cases in the RTX-KTx group and 28 (90.3%) of 31 cases in the C-KTx group developed HLA class II positive after KTx. The number of HLA class II dnDSA was significantly lower in the RTX-KTx group (*P* = .027). A few patients were diagnosed as both HLA class I and II positive (6.3% and 3.2%, respectively). No significant difference was found between the two groups in the mean dnDSA MFI levels in dnDSA-positive patients (*P =* .893).

**Table 4:** dnDSA production.

	RTX-KTx	C-KTx	
*N*	115	115	*P*
Total (%)	16 (13.9)	31 (26.9)	.005
Class I	0	2	.541*
A	0	2	
B	0	0	
Class II	15	28	.027
DR	2	5	
DQ	8	20	
DR + DQ	5	3	
Class I + II	1	1	1*
A + DQ	0	1	
B + DR + DQ	1	0	
MFI	8416±5517	8509±5504	.893

* Fisher’s exact test

MFI, mean fluorescence intensity

## Discussion

The complete depletion of T cells has been regarded as preventing allo-graft rejection and maintaining great renal function [23–27]. For that reason, we have specifically executed T cell depletion for ABO-compatible kidney transplantation (ABO-CKTx) to avoid acute cellular rejection by induction therapy and high-dose maintenance immunosuppression. Based on results of a large cohort study, Terasaki et al. reported in 2004 that 1-year graft survival in KTx recipients with anti-HLA antibodies was significantly worse than that in KTx recipients without anti-HLA antibodies [28]. During the last decade, some investigators have reported that B cell depletion by rituximab for ABO-incompatible living kidney transplantation (ABO-ILKTx) recipients might serve a crucially important role in suppression of DSA production and incidence of ABMR [12, 29]. Therefore, the use of rituximab for ABO-ILKTx and immunologically high-risk KTx is increasing. Tyden et al. first reported a randomized control trial of single-dose rituximab induction therapy (375 mg/m^2^ body) for ABO-CKTx recipients with a panel reactive antibody (PRA) less than 50% [19]. Results of follow-up studies conducted by the same group showed no significant benefit for rituximab-treated ABO-CKTx recipients in three-year graft and patient survival rates [20]. Hoogen et al. recently analyzed single-dose, intra-operative, rituximab induction (375 mg/m^2^ body) for 6 months, obtaining results suggesting that rituximab induction is a useful strategy in immunologically high-risk ABO-CKTx recipients [21]. Nevertheless, 45–70% kidneys included among these data were obtained from deceased donors. Results of five-year follow-up in the present study demonstrate that single fixed low-dose rituximab as induction therapy suppresses the incidence of BPAR and production of dnDSA in non-sensitized ABO-CLKTx recipients.

To avoid adverse events, minimized and modified immunosuppressive strategies have achieved great outcomes in preclinical transplant models. D’Addio et al. reported that prolonged low-dose ATG with CTLA4-Ig produced significant benefits for skin graft prolongation in rat transplantation [30]. Carvello M et al. also demonstrated that a new agent such as anti-CD22 monoclonal antibody conjugated with calicheamicin (anti-CD22/cal) was effective to deplete B cells and to suppress donor-specific immune response. Finally, murine treated with a combination therapy using anti-CD22/cal and CTLA4-Ig prolonged islet graft survival significantly [31]. These results suggest that we can develop low-dose rituximab induction therapy for suppressing dnDSA production and for avoiding ABMR in ABO-CLKTx.

As reported based on results of an earlier study [5], we demonstrated that 1-year renal function of ABO-ILKTx recipients with 100 mg dose rituximab induction was completely equivalent to that achieved with a 200 mg or 500 mg dose. Therefore, we chose to administer single low-dose rituximab for induction therapy by intravenous injection of 100 mg on day 4 before KTx with standard immunosuppression. Schroder et al. reported that B cells in peripheral blood were eradicated within 72 hr of rituximab administration [32]. That fact was consistent with our results of CD19 positive B cells in peripheral blood mononuclear cells at pre-KTx and post-KTx. By contrast, earlier reports presented by Tyden et al. and Hoogen et al. described that no improved graft survival or incidence of rejection was found in non-sensitized ABO-CKTx recipients with rituximab induction alone, but that it maintained standard immunosuppression [19, 21]. Rituximab had completely eradicated CD19 positive B cells and memory B cells for 12 months after transplantation, but it did not affect T cells [33]. Therefore, we chose B cell depletion by rituximab in addition to interleukin-2 receptor antagonist as induction for non-sensitized ABO-CLKTx recipients because T cell-mediated antigen presentation through B cells causes acute rejection and causes it more significantly in cases of chronic allo-graft injury.

Results of five-year follow-up revealed that graft survival tends to be better for the rituximab-treated group. Nevertheless, no significant difference was found. By contrast, we confirmed that induction therapy using single fixed low-dose rituximab on day 4 before KTx was sufficient to reduce the incidence of BPAR, especially TCMR. Agarwal et al. reported that high-dose rituximab induction (375 mg/m2 body) caused cytokine storm including interleukin-6 (IL-6) and tumor necrosis factor-α (TNF-α). However, their cytokine release was detected by transient increase with minimal changes [34]. By contrast, Joosten et al. showed that higher release of regulatory cytokines IL-10, but not other inflammatory cytokines such as IL-6, IL-17, TNF-α, and interferon-γ, was detected in serum at 2 hr after rituximab administration [35]. Most of the T cells showed Th2-like phenotype *in vitro*, although no decline in T cell proliferation was observed in B cells receiving rituximab administration. This phenomenon under *in vivo* settings has not been clarified. Follow-up studies of recipients who underwent BPAR after rituximab induction therapy indicated that CD79a positive and CD138 positive plasma cells were suppressed in peripheral blood mononuclear cells [36]. Considering these reported results together with our own, we infer that dual directed induction therapy for both B cells and T cells might be necessary to reduce the incidence of BPAR.

Results obtained from this study confirmed that rituximab plus interleukin-2 receptor antagonist as induction was sufficient to suppress the production of dnDSA after ABO-CLKTx. The presence of dnDSA negatively affects the incidence of AABMR and CABMR, leading to poor graft survival. Recently, several reports have described that AABMR is strongly attributable to the specific dnDSA, such as against HLA class II antibodies, MFI levels greater than 3000, and C1q status [14–17]. In fact, HLA class I antibodies had higher risk for AABMR than those with HLA class II antibodies. However, HLA class II antibodies were associated with CABMR [13]. We demonstrated that higher percentages of HLA class II antibodies and ABMR existed in rituximab-untreated recipients at five-year follow-up. By contrast, Yamamoto et al. reported that approximately 60% of the recipients with dnDSA maintained stable renal function for 1 year, with no development of ABMR [17]. Similar to this finding, only 20 (42%) of 47 recipients with dnDSA were found to have sustained any AABMR or CABMR in our study. Taken together, these results suggest that not all dnDSA caused development of ABMR.

As we had expected, the incidence of severe leukopenia was detected as approximately 1.5 times higher after rituximab induction. Nevertheless, our study results were insufficient to confirm any increase in rituximab-treated groups with regard to CMV and BKV infection, which is consistent with earlier observations [19, 21, 29]. We assumed that treatment for BPAR caused higher incidence of CMV infection in the group that was not treated with rituximab because CMV serologic status before KTx was similar in the two groups. This study demonstrated that patient survival in the rituximab-treated group is compatible with that of the control group: only 1 patient died of cerebrovascular disease. Some investigators have reported higher mortality in rituximab-treated patients attributable to myocardial infarction, cardiac arrest, and infectious disease [20, 37]. The B cells might have an atheroprotective role [38]. However, this role is explainable by the fact that patients in these studies have undergone excess B cell suppression for high doses (375 mg/m^2^ body) or multiple doses of rituximab treatment. We confirmed that a 100 mg single dose of rituximab infusion is safe for non-sensitized ABO-CLKTx recipients.

This study had several limitations. This retrospective observational study was conducted using data from a single center. Moreover, protocol biopsies were not obtained, possibly confounding data related to the respective incidences of subclinical ABMR and TCMR, particularly in patients that had developed dnDSA. Moreover, the follow-up of DSA value was insufficiently long to follow all cases for five years. Our study should be regarded as a pilot study assessing the risk of dnDSA and BPAR using rituximab induction for non-sensitized ABO-CLKTx recipients. A large-scale prospective study or a longitudinal study with longer than 10-year follow-up can be expected to yield useful results for precise evaluation of the risk factors affecting chronic allo-graft injury.

In conclusion, results of this study demonstrated that single fixed low-dose rituximab as induction therapy was sufficient for ensuring safety, reducing rejection, and suppressing dnDSA production for immunological low-risk non-sensitized ABO-CLKTx.

## Abbreviations

AABMR: acute/active antibody-mediated rejection
ABMR: antibody-mediated rejection
ABO-CKTx: ABO-compatible kidney transplantation
ABO-CLKTx: ABO-compatible living kidney transplantation
ABO-ILKTx: ABO-incompatible living kidney transplantation
ATG: antithymocyte globulin
BKV: BK virus
BPAR: biopsy-proven acute rejection
BXM: basiliximab
CABMR: chronic active antibody-mediated rejection
DFPP: double filtration plasmapheresis
DGF: delayed graft function
dnDSA: de novo donor-specific anti-human leukocyte antigen antibodies
DSA: donor-specific anti-human leukocyte antigen antibodies
eGFR: estimated glomerular filtration rate
ESRD: end-stage renal disease
HLA: human leukocyte antigen
MMF: mycophenolate mofetil
PRA: panel reactive antibodies
PSS: propensity score analysis
PTC: peritubular capillaries
TCMR: T cell-mediated rejection
